# Supplementary material for: Comparative Study of Vaccinated and Unvaccinated Hospitalised Patients: A Retrospective Population Study of 500 Hospitalised Patients with SARS-CoV-2 Infection in a Spanish Population of 220,000 Inhabitants
Source: Viruses. 2022 Oct 17;14(10):2284. doi: 10.3390/v14102284 (PMC9611498; doi:10.3390/v14102284)
Supplement: Supplementary file 1 [file viruses-14-02284-s001.zip › viruses-1962920-supplementary.pdf]

§ **Fuencovid study group:**

**Coding Department:** Maite Cobos

**Radiology:** Daniel Castellón Plaza, Gabriel Nombela Fernández, Blanca Gener Laquidáin, Elena Rodríguez Palacín, Carmen Díaz del Río Martínez, Carlos Alonso Hernández Rodríguez, Ana Vaca Barrios, Tamara Rodríguez Uribe, Rosa Sierra Torres, Marta González-Ruano Iriarte.

**Hospital Pharmacy:** Ana Beatriz Fernández Román, Ana Ontañón Nasarre, Aranzazu Pou ,Beatriz Candel García ,Belen Hernández,Carolina Mariño, Cristina Bravo Lázaro, Cristina Puivecino Moreno, Eva María García, Jorge Pedreira Bouzas, Maria del Mar García, Maria Jesús Esteban Gómez, Maria José Canalejo, Mario García Gil, Nuria Font, Nuria Herrero Muñoz, Paloma Gabaldón, Yolanda Castellanos Vicente.

**Clinical Laboratory:** Natalia González Pereira,Santiago Prieto Menchero

**Intensive Care Unit:** Angela Alonso Ovies, Joaquín Álvarez Rodríguez, Susana Blanco Pintado, Mª Ángeles de la Torre Ramos, Raquel del Olmo Monge, Melissa Echeverría León, Almudena Escribá Bárcena, Susana Godoy Boraita, Ana Gutiérrez García, María Lozano Espinosa, Beatriz Nieto Martino, Ana Isabel Rodríguez Villar, Teresa Saldaña Fernández, Carolina Sena Pérez, Eugenia Sevillano Pérez, Enrique Torres Sánchez, Clara Vaquerizo Alonso: Ferney Vasco Castaño, Carlos Velayos Amo, Gema Arellano del Verbo, Gema Díaz Cuero.

**Internal Medicine:** Aida Izquierdo Martínez, Alejandro Morales Ortega, Ana Farfan Sedano, Begoña Frutos Pérez, Carlos Enrique Suárez Acosta,Celia Lara Montes, Cristina De Ancos Aracil ,David Bernal Bello,Elena María Sáiz Lou , Guillermo Soria Fernández, Ibón Ayala Larrañaga, Jorge Marrero Francés, Jose Ángel Satué Bartolomé, Jose Manuel Ruiz Giardín, Juan Víctor Sanmartín López, Lorena Carpintero García, Luis Rivas Prado, Maria Inmaculada Salamanca, María Toledano Macías, Marta Guerrero Santillán, Marta Rivilla Jiménez, Miguel Ángel Duarte Millán, Miguel de Hita Castillo, Nieves Mesa-Plaza, Rafael Cristóbal Bilbao, Raquel Jiménez Ortego, Ruth Calderón, Sara Piedrabuena García, Sonia Gonzalo Pascua, Virginia Garcia Bermúdez, Alicia Moreno Borreguero, Emilia Cáncer Minchot,Irene Gonzalo Montesinoa Maria Rosa Villar Vicente, Soralla Civanos Modino, Eva Prats Graciá, Maria Rabasa Pérez

**Information Systems:** Angel Arias Cosin, Esther Pereira Herrero, Jorge Augusto Moreno, Jorge Tarancón Rey, Luis Antonio Lechuga Suárez, Vicente Aranzana González

**Emergency Deparment:** Nancy Geraldine Marquina Colacci, Sonsoles Callejas Pérez, Alberto Sánchez Bernal, Mónica Coll Hernández, Madelyn Vianessa Ramírez Reyes, Paloma Pardo Rovira, Elena Tejero Sánchez, Belén Mora Hernández, Mª Eugenia Barrero Ramos, Miriam Quero Escalada, María Isabel López Rodríguez, Lourdes Muñoz Abril, Gema Delgado Cárdenas, Laura Mariel Matus, Alexandre Maurice Sarradey, María José Durán Gallego, Patricia Sabio Repiso, Marta Álvarez Alonso, Sonia Sánchez Sánchez, Cristina Latorre Marco, Inmaculada Salamanca Ramírez, Kristina Kosanic Martín del Campo, Laura Sánchez Amo, Maria Jesús Domíguez García.

**Table S1.** Study variables *.

| Variable | Description | Units (International System), If Applicable |
|---|---|---|
| **Epidemiological Data** | | |
| Age | Patient's age at the time of admission | years |
| Gender | M or F | |
| Place of birth | Caucasian, Sub-Saharan, North African, Asian, Latin American. | |
| Nursing home | Patient comes from a nursing home yes or not | |
| **Medical history** | | |
| BMI | Measure of body fat that results from dividing the weight by the height | $kg/m^2$ |
| HBP | Yes or not | |
| DM | Diabetes mellitus (any type) yes or not | |
| Cardiopathy | Ischemic heart disease or heart failure yes or not | |
| Smoker | Active smoker until admission | |
| COPD | Yes or not | |
| Asthma | Yes or not | |
| Hematological disease | Any malignant haemotological disease | |
| Oncological disease | Any solid oncological disease | |
| Autoimmune disease | Any | |
| HIV | Confirmed HIV infection: yes or not | |
| Dementia | Yes or not | |
| Charlson | Charlson comorbidity index: predicts 10-year survival in patients with multiple comorbidities. | |
| Type of vaccine | Type of vaccine received against SARS-CoV-2 infection: BNT162b2 mRNA (Pfizer/BioNTech), mRNA-1273 (Moderna), ChAdOx1 nCoV-19/AZD1222 (AstraZeneca/Oxford), Ad26.COV2.S (Janssen) | |
| Vaccination | Vaccinated:<br>- BNT162b2 mRNA: two-dose schedule 21 d apart.<br>- mRNA-1273: two-dose schedule 28 d apart.<br>- ChAdOx1 nCoV-19/AZD1222: two-dose schedule 56-84 d apart.<br>- Ad26.COV2.S: single dose.<br>14 d period after second dose is required to considered patient complete vaccinated.<br>Not vaccinated: patient has not received any vaccine dose or received an incomplete schedule or after receiving the complete schedule started with COVID-19 symptoms before 14 d apart from de last dose | |
| **Radiological findings** | | |
| Initial chest radiography | No pneumonia<br>Unilateral pneumonia<br>Bilateral pneumonia | |
| **Clinical evolution** | | |
| Severity | Severity of COVID-19 infection classified at discharge<br>Mild: no pneumonia<br>Moderate: pneumonia in the absence of severity criteria<br>Severe: oxygen saturation less than 93%, tachypnea or radiological extension of pneumonia greater than 50% | |
| Highest oxygen requirements | Highest oxygen support needed during admission in the hospital ward (ICU not included): not oxygen, VM 28%, VM 31%, VM 35%, VM 50%, NRBM 100% | |
| ICU admission | Yes/No | |
| Death in hospital | Yes/No | |

| | | |
|---|---|---|
| Hospital stay | From hospital admission to discharge | days |
| ICU stay | From ICU admission to discharge | days |
| Death at one month | Wether the patient is death one month after the discharge or not | |
| **Laboratory test** | | |
| Highest CRP | Highest CRP value during admission | mg/dL |
| Highest IL-6 | Highest IL-6 value during admission | pg/mL |
| Highest IL-6 pre-treatment with tocilizumab | Highest IL-6 pre-treatment with tocilizumab during admission | pg/mL |
| Lowest lymphocyte value | Lowest lymphocyte value during admission | lymphocytes/mcL |
| Highest DD | Highest DD value during admission | ng/mL |
| Highest LDH | Highest LDH value during admission | ng/mL |
| Highest ferritin | Highest ferritin value during admission | ng/mL |
| **Treatments** | | |
| Remdesivir | Wether the patient received it or not | |
| Tocilizumab | Wether the patient received it or not | |
| Imatinib | Wether the patient received it or not | |
| Baricitinib | Wether the patient received it or not | |
| Anakinra | Wether the patient received it or not | |
| Low-molecular-weight heparin | Wether the patient received it or not | |
| Corticosteroids | Wether the patient received it or not | |

*BMI: body mass index; HBP: high blood pressure; DM: diabetes mellitus; COPD: chronic obstructive pulmonary disease; HIV: human immunodeficiency virus; SARS-CoV-2: severe acute respiratory syndrome coronavirus 2; ICU: intensive care unit; VM: venturi mask; NRBM: non-rebreather mask; CRP: C-reactive protein; IL-6: interleukin-6; DD: D-dimer; LDH: lactate dehydrogenase.

**Table S2.** Type of vaccine in the fully vaccinated cohort*.

| Vaccine | Vaccinated |
|---|---|
| BNT162b2 mRNA (Pfizer/BioNTech) | 61 (79.2) |
| mRNA-1273 (Moderna) | 3 (3.9) |
| ChAdOx1 nCoV-19/AZD1222 (AstraZeneca/Oxford) | 7 (9.1) |
| Ad26.COV2.S (Janssen) | 6 (7.8) |
| Total | 77/77 (100.0) |

*Values are no. (%).
